# Cytometric analysis of adipose tissue reveals increments of adipocyte progenitor cells after weight loss induced by bariatric surgery

**DOI:** 10.1038/s41598-018-33488-7

**Published:** 2018-10-12

**Authors:** Jesús García-Rubio, Josefa León, Anaïs Redruello-Romero, Esther Pavón, Antonio Cozar, Francisco Tamayo, Mercedes Caba-Molina, Javier Salmerón, Ángel Carazo

**Affiliations:** 1 Surgery Unit, San Cecilio University Hospital, Granada, Spain; 2 ibs.Granada (Instituto de Investigación Biosanitaria), Granada, Spain; 3 Ciberehd (Centro de Investigación Biomédica en Red de Enfermedades Hepáticas y Digestivas), Granada, Spain; 4 Clinical Management Unit of Digestive System, San Cecilio University Hospital, Granada, Spain; 5 Pathological Anatomy Unit, San Cecilio University Hospital, Granada, Spain; 6 Medicine Department, University of Granada, Granada, Spain

## Abstract

Obesity-related comorbidities are, in large part, originated from the dysfunction of adipose tissue. Most of them revert after the normalization of body mass. Adipose tissue is essentially occupied by adipocytes. However, different populations of immunological cells and adipocyte precursor cells (AdPCs) are the main cellular components of tissue. During obesity, body fat depots acquire a low-level chronic inflammation and adipocytes increase in number and volume. Conversely, weight loss improves the inflammatory phenotype of adipose tissue immune cells and reduces the volume of adipocytes. Nevertheless, very little is known about the evolution of the human AdPCs reservoir. We have developed a flow cytometry-based methodology to simultaneously quantify the main cell populations of adipose tissue. Starting from this technical approach, we have studied human adipose tissue samples (visceral and subcutaneous) obtained at two different physiological situations: at morbid obesity and after bariatric surgery-induced weight loss. We report a considerable increase of the AdPCs reservoir after losing weight and several changes in the immune cells populations of adipose tissue (mast cells increase, neutrophils decrease and macrophages switch phenotype). No changes were observed for T-lymphocytes, which are discussed in the context of recent findings.

## Introduction

The incidence of obesity is rising dramatically. This in turn increases the risk of developing type II diabetes, fatty liver disease and cardiovascular disease. It is also associated with other pathologies such as cancer, immune disorders and psychiatric disturbances^1–3^. As a consequence of both, increasing incidence and wide spectrum of comorbidities, obesity is currently considered a major public health problem. Most of the obesity-related pathological processes revert after the normalization of body mass^4^. However, in obese subjects, weight reduction by voluntary caloric restriction is habitually followed by a new cycle of body mass regain^5^. At present, bariatric surgery remains the most effective treatment for permanent weight loss^6^.

During the last decade, it has emerged the concept that obesity-related comorbidities are, in large part, originated in the adipose tissue itself^7,8^. Continuous expansion of body fat depots has a great impact on adipose tissue remodelling and function^9^. At cellular level, adipose tissue cells acquire a chronic proinflammatory phenotype^10,11^ and adipocytes increase in number and volume^12,13^. At functional level, adipose tissue alters the profile of released hormones and cytokines^14,15^. Moreover, according to certain hypotheses^9^, it limits the ability for further adipose tissue enlargement.

Adipose tissue is essentially occupied by adipocytes but there are other types of cells – often in large quantities^16,17^. Immunological cells (from both, innate and adaptive immune systems) and different populations of adipocyte precursor cells (AdPCs) are the main components of the stromal vascular fraction (SVF) of adipose tissue. The state of low-level chronic inflammation, promoted by obesity at fat depots, begins to reverse a few months after surgery-induced weight loss^18^. Concerning AdPCs, only few studies have estimated its pool *in vivo*. Furthermore, there are no standardised methods to identify and quantify human AdPCs. As a result, very little is known about the evolution of the human AdPCs reservoir under different physiological situations.

We performed a flow cytometry-based methodology to simultaneously measure the main cell types in SVF. This study aims to explore tissue remodelling, at cell level, in the context of weight loss induced by bariatric surgery.

## Methods

### Patient cohorts

The ethics committee of the *Portal de Ética de la Investigación Biomédica de Andalucía* approved the study and it was conducted according to the guidelines approved by the committee. All patients provided written informed consent for sampling and publication. All patients’ names were encoded by the hospital to remove any trace of patient identity. In this study, we analysed two different cohorts. On the one hand, the *morbidly obese patient cohort* included 43 patients who underwent laparoscopic bariatric surgery (14 gastric bypass and 29 gastric sleeve). On the other hand, the *ex-morbidly obese patient cohort* included 28 ex-morbidly obese subjects after significant weight loss (between 35 and 70 Kg), and after an elapsed time ranged from 12 to 18 months since bariatric surgery. All ex-morbidly obese patients underwent abdominoplastic surgery to remove the excess of abdominal skin. In addition, in 21 of them, wall hernias (originated from previous bariatric surgeries) were also repaired through the abdominoplasty. The surgical interventions were performed at San Cecilio University Hospital of Granada (Spain).

### Biological samples

Blood samples were obtained at the moment of surgery. Adipose tissue biopsies were obtained from two different fat depots with several variations depending on the surgical procedure. In laparoscopic bariatric surgeries, visceral adipose tissue biopsies were obtained at the greater omentum, near the stomach, whereas subcutaneous adipose tissue biopsies were obtained at the area of the surgical incision. In abdominoplastic surgeries, subcutaneous tissue biopsies were collected from removed skin, whereas visceral tissue biopsies were obtained thought the abdominal wall eventrations.

### Biochemical parameters

Blood samples were processed and analysed by routine methods within 24 h at the Clinical Analysis Laboratory of San Cecilio University Hospital (Granada, Spain). The model assessment (HOMA-IR) index was calculated to evaluate insulin resistance.

### Histology of adipose tissue samples

A fraction of adipose tissue biopsies were fixed and prepared for histologic analysis by using standard procedures. The adipocyte size (μm) was estimated by measuring the major diameter of 200 cells from digital microscopic images and using Image J software (NIH-Bethesda).

### Isolation of the stromal vascular fraction (SVF) from adipose tissue samples

Immediately after surgical extraction, adipose tissue biopsies were preserved on ice, in a physiological buffered solution (Dulbeccos’s PBS). Visible blood vessels were remove from biopsies. In addition, visceral adipose tissue samples were carefully examined to identify and remove small lymph nodules eventually present in visceral depots. Two grams of adipose tissue were cut in small pieces and enzymatically disaggregated by the application of 10 mg of collagenase I (Sigma) in 6 ml of RPMI 1640 medium supplemented with 5 mM CaCl_2_, at 37 °C during 90 minutes. After enzymatic treatment, samples were diluted with 40 ml of Dulbeccos’s PBS, passed through a sieve with 1 mm mesh and centrifuged at 750 × g for 10 minutes in order to separate SVF (pellet) from adipocytes and released lipids (supernatant). SVF cells were resuspended in 10 ml of Dulbeccos’s PBS, filtered through a 100 µm mesh sieve, centrifuged and, finally, resuspended in 500 µl of antibody staining buffer (Dulbeccos’s PBS, 2% fetal bovin serum, 0.09% albumin, 0.05% sodium azide) in which an internal standard (TrueCount^TM^ BD Biosciences) was previously reconstituted.

### Antibody staining and flow cytometry

Freshly isolated SVF cells were incubated with fluorescent-conjugated antibodies or their respective controls during 20 minutes at room temperature. Then, cells were resuspended during 30 minutes in BD FACS^TM^ Lysin Solution in order to fix cells and lyse erythrocytes. After a wash step, they were preserved at 4 °C in staining buffer. Data acquisition was performed using a FACS ARIA II flow cytometer (FACSDiva software v5) equipped with two lasers (blue and red) within 24 h after SVF isolation. Moreover, to estimate the cell death ratio, an aliquot of SVF was incubated with anti-CD34 APC-conjugated and 10 μg/mL propidium iodure (which stains only nonviable cells) and immediately analysed by flow cytometry without fixation. Samples with death ratio upper than 10% were excluded from the study.

Compensation beads, isotype controls and fluorescent-conjugated antibodies were purchased from BD Biosciences and BioLegend. All antibodies were mouse monoclonal and specific against human cell surface markers. Fluorescent-conjugated antibodies were grouped in four analytical panels, which were carefully designed taking into account the cytometer configuration and the peculiarities of tissue cytometry. Panel I (AdPCs and endothelial cells): anti-CD45 PE-CF594-conjugated (clone HI30, BD), anti-CD34 APC-conjugated (clone 581, BD) and anti-CD31 APC-Cy7-conjugated (clone WM59, BD). Panel II (neutrophil and macrophages): anti-CD45 PE-CF594-conjugated clone HI30, BD), anti-CD15 PE-conjugated (clone HI98, BD), anti-CD14 FITC-conjugated (clone M5E2, BD) and anti-CD11c APC-conjugated (clone B-ly6, BD). Panel III (mast cells): anti-CD45 PE-CF594-conjugated (clone HI30, BD), anti-CD14 FITC-conjugated (clone M5E2, BD), anti-CD117 APC-conjugated (clone YB5.B8, BD) and anti-FcεRIα PE-Cy7-conjugated (clone AER-37, BioLegend). Panel IV (T-lymphocytes): anti-CD45 PE-CF594-conjugated (clone HI30, BD), anti-CD3 APC-Cy7-conjugated (clone SK7, BD), anti-CD8 APC-conjugated (clone RPA-T8, BD), anti-CD4 BB-515-conjugated (clone SK3, BD) and anti-CD19 BB-515-conjugated (clone HIB19, BD). Cell populations analysed in the study were identified by the following patterns of surface markers. AdPCs**:** CD45−/CD34+/CD31−. Endothelial cells: CD45−/CD34+/CD31+. Neutrophil: CD45+/CD15+/CD14− (or CD14^low^). Macrophages: CD45−/CD15−/CD14+(CD11c as proinflammatory marker). Mast cells: CD45+/CD14−/CD117+/FcεRIα+. T-helper lymphocytes: CD45+/CD3+/CD4+/CD8−. T-cytotoxic lymphocytes: CD45+/CD3+/CD4−/CD8+. B-lymphocyte: CD45+/CD3−/CD19+.

### Statistical analysis

Continuous variables are expressed as means ± standard deviation and categorical variables are expressed as numerals. Continuous variable distributions were assessed for skewness and, when found, they were logarithmically transformed for all analyses. An independent t-test was used to compare differences between means. Within-patient changes from visceral to subcutaneous fat were analysed by paired t-tests. Differences associated to weight loss were assessed by non-paired t-test. The presence of multicollinearity in multivariate models was evaluated using the variance inflation factor, with a value > 5 suggesting its absence. The data were analysed using the SPSS program for Windows, version 22.0 (IBM Corp., Armonk, NY). A p-value < 0.05 was considered as statistical significant.

## Results

### Baseline in patient cohorts

Table 1 shows the average data for age, gender, BMI and biochemical parameters of the two cohorts analysed in this work, morbidly obese and ex-morbidly obese patients. A characteristic of our study is the predominance of women (around 65% in both cohorts) due to social constraints in our geographic environment. Although subjects of the ex-morbidly obese cohort remain within the range considered as obesity (average BMI of 31.8 ± 1.2), we can observe the improvement of biochemical parameters and type II diabetes prevalence. This suggests that patients included in the ex-morbidly obese cohort have started the reversion from obesity-related metabolic disturbances.

**Table 1:** Baseline characteristics.

Characteristic	Group		p
	Morbidly Obese (N = 43)	Ex-Morbidly Obese (N = 28)	
Age (years)	43.8 ± 9.7	46.8 ± 9.0	0.197
Male/Female	15/28	11/17	0.803
Type II diabetes	15 (34.8%)	2 (7.1%)	**0.010**
BMI (kg/m^2^)	48.5 ± 1.5	31.8 ± 1.2	**<0.001**
Glucose (mg/dl)	105.4 ± 1.4	82.6 ± 1.2	**0.001**
Insulin (units/ml)	9.5 ± 7.3	2.1 ± 0.9	**0.005**
HOMA	2.8 ± 1.8	0.5 ± 0.1	**0.052**
C-peptide (ng/ml)	3.2 ± 1.4	1.2 ± 0.3	**<0.001**
HbA1c (%)	6.0 ± 1.2	5.2 ± 1.1	**<0.001**
Urea (mg/dl)	30.0 ± 10.9	28.8 ± 9.7	0.830
Uric Acid (mg/dl)	6.2 ± 1.9	4.3 ± 1.7	**<0.001**
Total Bilirubin (mg/dl)	0.5 ± 0.3	0.5 ± 0.2	0.712
AST (units/l)	45.0 ± 29.5	27.0 ± 9.9	0.406
ALT (units/l)	34.7 ± 1.8	19.2 ± 1.8	**0.001**
GGT (units/l)	33.5 ± 2.2	17.8 ± 2.3	**0.005**
Alkaline Phosphatase (units/l)	67.0 ± 23.8	84.7 ± 22.9	**0.010**
Total Creatinine Kinase (units/l)	113.9 ± 79.9	107.1 ± 68.7	0.814
Amylase (units/l)	39.6 ± 11.9	51.6 ± 21.7	0.072
Cholesterol (mg/dl)	157.2 ± 32.0	152.0 ± 54.1	0.632
Triglycerides (mg/dl)	158.2 ± 52.8	87.7 ± 31.3	**<0.001**
HDLc (mg/dl)	37.3 ± 1.3	48.0 ± 1.4	**0.013**
LDLc (mg/dl)	86.2 ± 30.5	87.5 ± 45.5	0.904
Iron (µg/dl)	75.6 ± 31.9	58.8 ± 32.6	0.075
Albumin (g/dl)	3.8 ± 0.3	3.6 ± 0.5	**0.042**
Sodium (mEq/l)	139.1 ± 3.1	141.2 ± 2.3	**0.015**
Potassium (mEq/l)	4.6 ± 1.5	4.3 ± 1.2	0.500

### Description of the flow cytometry strategy

We have develop a flow cytometry-based methodology in order to simultaneously identify and quantify seven SVF populations: AdPCs, endothelial cells, neutrophils, macrophages, mast cells, T-lymphocytes and B-lymphocytes.

Two modifications were introduced in the protocol for SVF isolation. Firstly, the enhanced digestion of tissue fibres increased the rate of cell extraction with minimal loss of cell viability, which remained higher than 90%. Only a few remains of fibres were visible on the mesh after enzymatic disaggregation of adipose tissue. As an additional advantage, our procedure reduced the contamination from tissue debris, allowing a better definition of cell populations during the cytometric analysis. Secondly, we have introduced an internal standard, composed of a known number of micrometric autofluorescent beads (with wide emission spectra from 600 nm). They can be differentiated from cell populations by measuring size (FSC, forward-scattered light) and complexity (SSC, side-scattered light). This procedure permits us to compare the data obtained among different flow cytometry experiments performed from the same sample.

**Figure 1:**
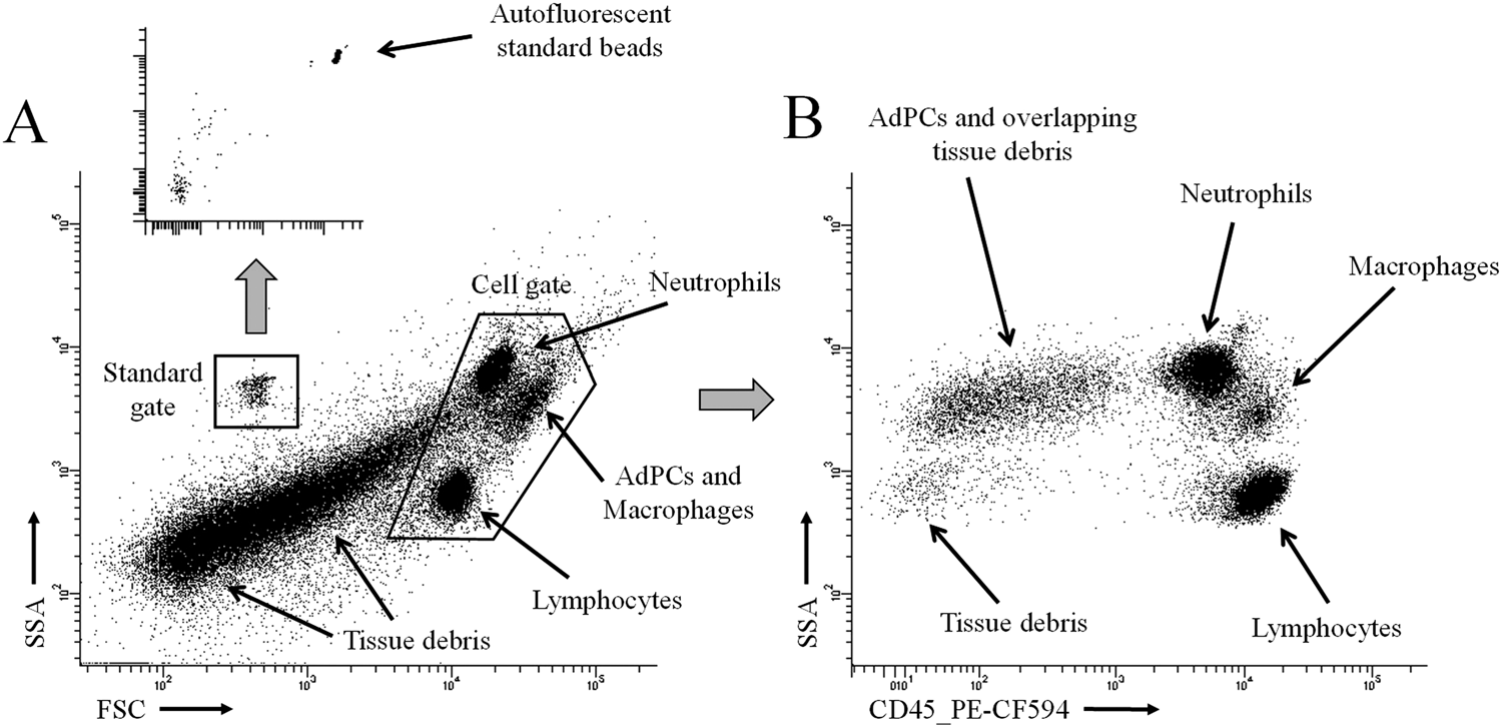
Common steps in the analysis of flow cytometry panels. Areas containing AdPCs, neutrophils, macrophages and lymphocytes (which are subsequently identified in four cytometry panels) are pointed out with arrows. (**A**) FSC-SSC plot, in which the gates for standards and cells are delimited. Autofluorescent standard beads are subsequent identified in two red channels. (**B**) Identification of immune cells (CD45+) from the cell gate.

Figure 1 shows the two common starting steps in the analysis of flow cytometry panels, which include the drawing of initial gates on the FSC-SSC plot and the identification of immune cells (CD45 is used as pan-leukocyte marker). Areas containing the most abundant SVF populations: neutrophils, lymphocytes and AdPCs (overlapped with macrophages) are visible on FSC-SSC plots (Fig. 1). A substantial proportion of cytometry events come from tissue debris. They are usually more abundant in subcutaneous adipose tissue and, likewise, may change among patients. The abundance of tissue debris probably depends on the degree of tissue fibrosis.

**Figure 2:**
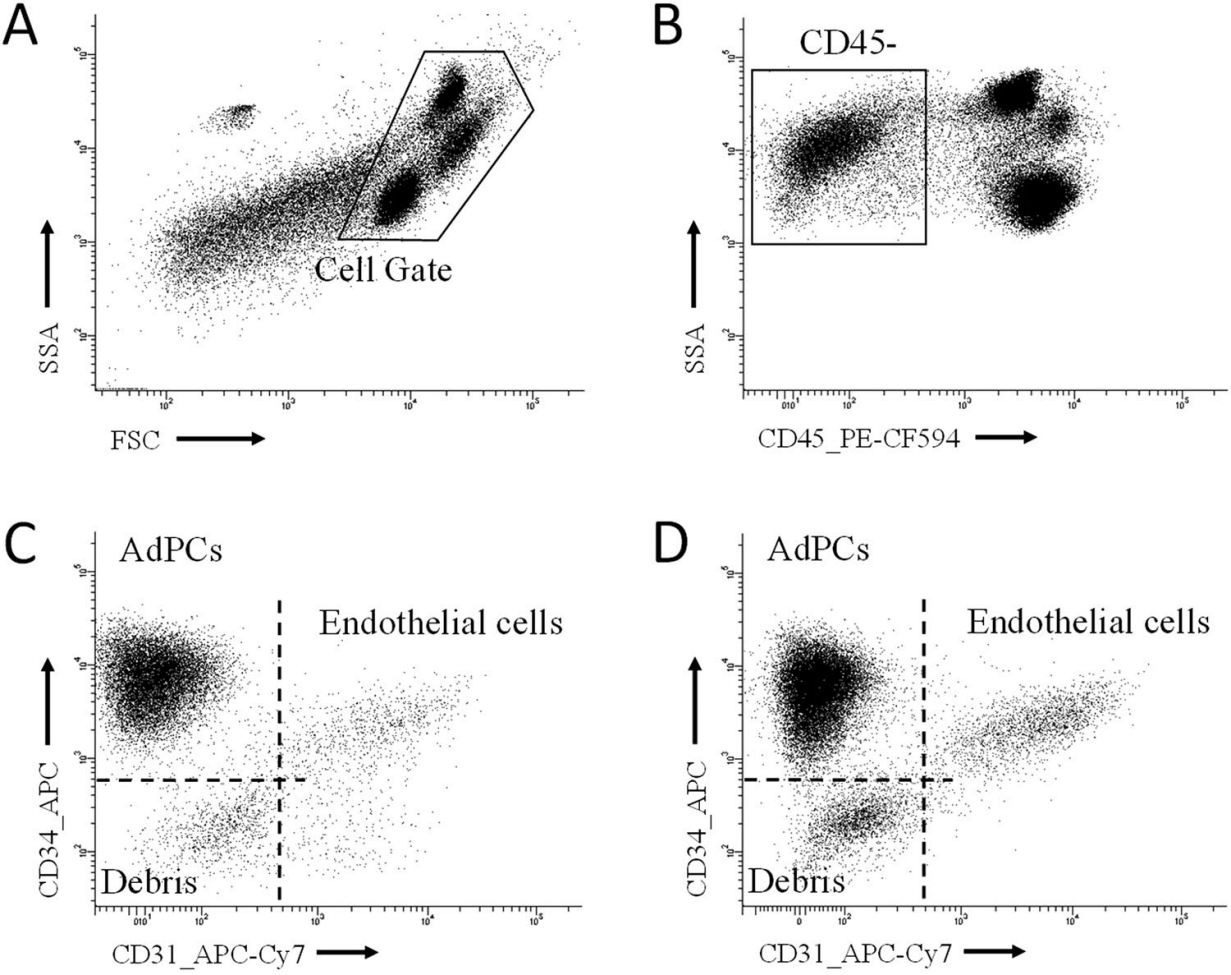
Sequential gating to identify AdPCs in panel 1. (**A**) Delimitation of cell gate in the FSD-SSC plot, (**B**) Identification of CD45− cells. (**C**) Identification of AdPCs (CD45−/CD34+/CD31−) and endothelial cells (CD45−/CD31+). Events negative for CD34 and CD31 come majorly from tissue debris. A, B and C belong to the same sample (visceral adipose tissue of a morbid obese patient). (**D**) Identification of AdPCs and endothelial cells from another sample (subcutaneous adipose tissue of a morbid obese patient).

In mice, the set of adipocyte progenitor populations are well characterised by a pattern of absence-presence of surface markers (CD45−/CD31−/CD34+/Sca1+/CD140a+)^17,19^. On the other hand, humans lack such a well-defined pattern and the most commonly used marker profile (CD45−/CD31−/CD34+) may be contaminated with stromal cells without adipogenic potential. Nonetheless, this latter fraction is composed of more than 95% of adipocyte progenitor cells in mice^20^, suggesting a similar proportion in humans. In order to simplify the terminology, the CD45−/CD31−/CD34+cytometry population is henceforth considered as human adipocyte progenitor cells (AdPCs). Meanwhile, endothelial cells can be identify by using the same markers but maintaining CD45−/CD31+ cells. Figure 2 shows the sequential gating to identify AdPCs and endothelial cells in panel I.

**Figure 3:**
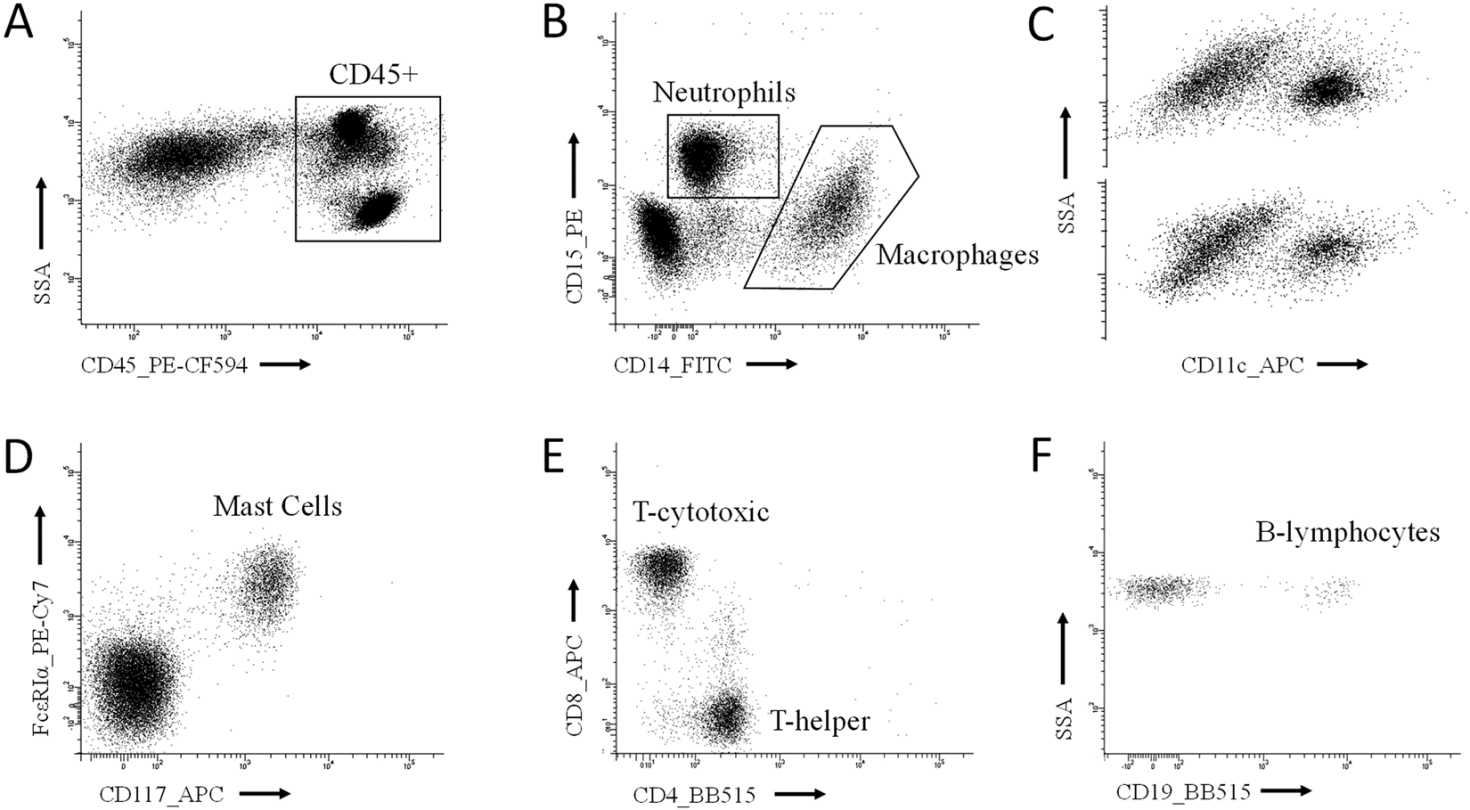
Sequential gating to identify immune populations. (**A**) All panels started from the identification of immune cells (CD45+) from the cell gate. (**B**) Panel II. Identification of neutrophils (high-SSC/CD45+/CD15+/CD14−) and macrophages (CD45+/CD15−/CD14+). (**C**) Two examples of the phenotypic identification of pro-inflammatory macrophages (CD11c+) from the macrophage gate. (**D**) Panel III. Identification of mast cells (CD117+/FcεRIα+) from a previous CD45+/CD14− gate (not showed). (**E**) Panel IV. Identification of T-helper (CD45+/CD3+/CD4+/CD8−) and T-cytotoxic (CD45+/CD3+/CD4−/ CD8+) from a previous CD45+/CD3+gate (not showed). (**F**) Panel IV. Identification of B-lymphocytes (CD45+/CD3−/CD19+) from a previous CD45+/CD3− gate (not showed).

Figure 3 explain the sequential gating to identify five immune populations (neutrophils, macrophages, mast cells, B- and T-lymphocytes) and three phenotypic subsets (CD11c+for macrophages and CD4+and CD8+ for T-lymphocytes) in panels II, III and IV. The group “other CD45+ cells” is composed by cells positive for CD45 but not identified as neutrophils, macrophages, mast cells, B- or T-lymphocytes. This group was lower than 5% and did not change between patient cohorts. These events were normally located in the lymphocyte gate, so they are likely to be natural killers. Nevertheless, in a few visceral adipose tissue samples we noted, just above neutrophils, a small population of eosinophils (high-SSC/CD45+/CD14+) which was included into “other CD45+ cells”. Due to the autofluorescent nature of macrophages in certain channels, mast cells were identified from a CD45+/CD14− gate in panel III. We note that CD4 and CD19 are conjugated with the same fluorochrome (BB515) and, however, they are used in panel IV. This is possible because both markers cannot cohabitate in the same cell.

**Figure 4:**
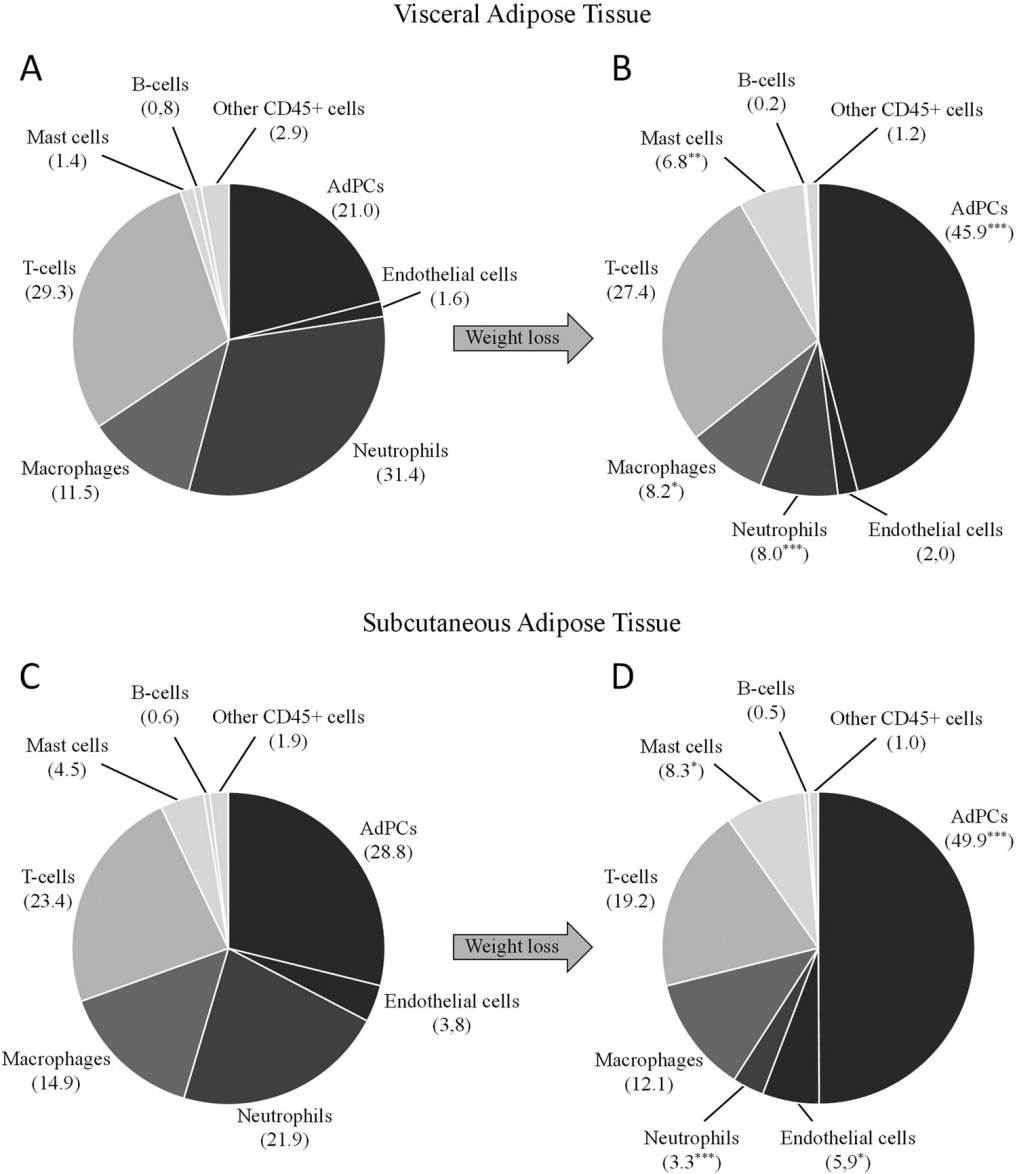
Cell composition of SVF. Data are expressed in percentage of cells within SVF, which was estimated by summing events positive for CD34 and CD45. ***P < 0.001, *P < 0.05 when comparing data between both types of patients. (**A**) Visceral adipose tissue of the morbidly obese cohort. (**B**) Visceral adipose tissue of the ex-morbidly obese cohort. (**C**) Subcutaneous adipose tissue of the morbidly obese cohort. (**D**) Subcutaneous adipose tissue of the ex-morbidly obese cohort.

Concerning the analysis of cytometry data, several concepts must be clarified before the formal exposition of results. Cells quantifications were expressed in absolute units (cells per grams of processed adipose tissue) and relative units (cell proportion within the “reference population”). For each sample, the reference population was defined by summing the ensemble of events positive for CD31, CD34 or CD45. These surface markers are expressed by the large majority of SVF cells. This approach permitted a proper estimation of the overall SVF without artificial interferences of overlapping tissue debris. One of the first things to consider is that most of variables expressed in cells/g followed an abnormal distribution. Conversely, data expressed in relative units (Fig. 4) usually followed normal distributions.

Relative units are commonly used for the analysis of flow cytometry data. However, they are sensitive to biological changes in the reference populations. Such is the case with the comparison between visceral and subcutaneous adipose tissues, which differ in the relative composition within the reference population and the bulk of SVF. Differently, absolute units allow cytometry data analysis without regard of the reference population, but are sensitive to mass variation of adipose tissue after weight loss. Based on the average reduction of adipocyte diameter in ex-morbidly obese patients (around 25%) and assuming a cell spherical shape, the decrease of adipocyte volume may be estimated between 50 to 60%, which is sufficient to double the density of estromal populations. To compare data expressed in absolute units between morbidly obese and ex-morbidly obese patients, we only took into account differences upper than two-fold.

### The reservoir of AdPCs decreases in morbidly obese patients with type II diabetes

It is known that the quantity of adipose tissue precursor cells may decrease in patients with type II diabetes^21^. There were 15 patients with type II diabetes in the morbidly obese cohort (Table 1). We have analysed the influence of type II diabetes on the seven SVF populations of this cohort. The presence of type II diabetes was related with a significant reduction of AdPCs from visceral adipose tissue, only when data were expressed by absolute units (Table 2). The level of decrease (together with the small number of diabetic patients) was not enough to be translated into a significant decrease of relative units (Table 2). No differences were observed for the remaining SVF populations.

**Table 2:** Diabetes-related serum variables and cytometry results for AdPCs.

Characteristic	Group		p
	No diabetes (N = 28)	Type II diabetes (N = 15)	
Male/Female	9/19	6/9	0.740
Glucose (mg/dl)	88.4 ± 1.5	133.5 ± 5.2	**<0.001**
Insulin (units/ml)	6.2 ± 1.2	14.6 ± 3.3	**<0.001**
HOMA	1.4 ± 0.7	3.7 ± 0.4	**0.020**
HbA1c (%)	5.6 ± 0.1	7.0 ± 0.4	**0.009**
Visceral AdPCs^a^	223.4 (103.4–293.1)	86.7 (58.8–167.1)	**0.031**
Subcutaneous AdPCs^a^	64.8 (34.2–101.0)	50.2 (29.0–133.4)	0.931
Visceral AdPCs (relative)^b^	23.7 ± 1.5	18.2 ± 1.5	0.071
Subcutaneous AdPCs (relative)^b^	29.0 ± 1.5	28.3 ± 1.5	0.757

^a^AdPCs in absolute units (10^3^ cells/g of adipose tissue). Data are presented as median ± interquartile range. ^b^AdPCs in relative units. Data are presented as percentage within the reference population (events positive for CD31, CD34 or CD45).

### The reservoir of AdPCs increases after surgery-induced weight loss

We observed a substantial increase of AdPCs quantity in the ex-morbidly obese patient cohort (Table 3 and Fig. 4). AdPCs is one of the main populations in morbidly obese patients but, after weight loss, it becomes the most abundant population of SVF (46% in visceral and 50% in subcutaneous adipose tissue). This increase is also appreciable in absolute units (five-fold in visceral and three-fold in subcutaneous tissue) even assuming an adipocyte volume loss around 50–60%.

**Table 3:** Adipocyte size and cytometric results expressed in absolute units.

	Visceral Adipose Tissue			Subcutaneous Adipose Tissue		
	Morbidly Obese	Ex-Morbidly Obese	p	Morbidly Obese	Ex-Morbidly Obese	p
Adipocyte Size^a^	167 (157–181)	128 (118–135)	**<0.001**	185 (172–194)^***^	141 (127–155)^##^	**<0.001**
SVF^b^	776 (469–1076)	1905 (1315–3093)	**<0.001**	240 (137–471)^***^	296 (222–613)^###^	0.035
Endothelial cells	10.1 (7.2–19.3)	28.8 (20.9–50.2)	**<0.001**	8.3 (5.1–14.4)^*^	20.3 (12.1–28.5)^##^	**<0.001**
AdPCs	155 (79–265)	956 (687–1226)	**<0.001**	56 (32–121)^***^	203 (80–311)^###^	**<0.001**
Neutrophils	221 (100–340)	83 (13–238)	**0.021**	36 (21–72)^***^	11 (6.4–19)^###^	**<0.001**
Macrophages	82 (55–140)	147 (82–303)	**0.053**	30 (19–63)^***^	35 (15–80)^###^	0.871
CD11c-Macrophages	41 (22–58)	40 (33–162)	0.190	18 (11–38)^***^	10 (5.1–16)^###^	**0.003**
% of CD11c^c^	45.4 (30.5–59.1)	35.6 (28.2–42.0)	**0.036**	58.8 (45.5–70.0)^***^	32.0 (21.1–35.1)	**<0.001**
Mast cells	9.9 (5.4–21)	97 (56–120)	**<0.001**	6.3 (3.6–14.1)^**^	25 (13–55)^###^	**<0.001**
T-Lymphocytes	233 (132–356)	379 (287–786)	**<0.001**	53 (28–94)^***^	60 (37–102)^###^	0.370
T-helper	97 (73–164)	120 (83–259)	0.342	31 (16–58)^***^	34 (22–52)^##^	0.497
T-cytotoxic	113 (48–161)	211 (109–246)	**0.002**	13 (92–27)^***^	17 (10–34)^###^	0.386
% of T-cytotoxic^d^	47.0 (36.7–51.1)	59.9 (41.9–61.0)	0.375	30.1 (25.2–34.9)^***^	32.2 (22.9–43.0)^#^	0.428
B-Lymphocytes	7.0 (2.9–13.0)	4.4 (2.2–10.4)	0.511	1.6 (0.7–3.7)^***^	1.2 (0.6–2.6)^###^	0.500

Data are represented as median ± interquartile range. ^a^Adipocyte size shows the mean of cell diameter in µm. ^b^SVF was estimated by summing events positive for CD31, CD34 or CD45. ^c^Percentage of the CD11c+phenotype within macrophages. ^d^Percentage of T-cytotoxic phenotype within T-lymphocytes. The remaining variables show 10^3^ cells/g of adipose tissue. ^***^P < 0.001, ^**^P < 0.01, ^*^P < 0.05 when comparing both types of adipose tissue in morbidly obese patients. ^###^P < 0.001, ^##^P < 0.01, ^#^P < 0.05 when comparing both types of adipose tissue in ex-morbidly obese patients.

**Table 4:** Multivariable linear regression analyses.

Variables	Visceral Tissue		Subcutaneous Tissue	
	β^b^	p	β^b^	p
Sex^a^	−0.084	0.467	−0.080	0.339
Age	0.013	0.915	−0.020	0.814
AdPCs	0.644	**<0.001**	0.435	**<0.001**
CD11c-Macrophages			−0.526	**<0.001**

Type of patient (morbidly obese versus ex-morbidly obese patients) is the dependent variable. ^a^Women are the reference group. ^b^β: Standardized coefficient. Multivariable analysis was conducted with the following variables: sex, age, proportion of AsPCs, proportion of CD11c+ phenotype within macrophages, proportion of T-cytotoxic phenotype within T-lymphocytes.

### Changes in endothelial cells after surgery-induced weight loss

In our study, the CD45−/CD31+ population (considered as endothelial cells) also showed low- or medium- expression level for CD34 (Fig. 2). However, in certain samples, a small population of CD45−/CD34−/CD31+ can be found (Fig. 2C). Endothelial cells represent a small fraction of the total amount of SVF (Table 3 and Fig. 4). We showed a modest increase in quantity (expressed in absolute units) after losing weight (Table 3). This increase (2.8-fold in visceral and 2.4-fold in subcutaneous adipose tissue) was barely enough to compensate an adipocyte volume loss; around 50–60% in ex-morbidly obese patients. When data were expressed in relative units, a significant increase was observed only in subcutaneous adipose tissue (Fig. 4).

### Changes in the immune cell populations after surgery-induced weight loss

Neutrophils and mast cells showed the largest change after losing weight – neutrophils decreased while mast cells incremented (Table 3 and Fig. 4). The increase of mast cells was remarkable in absolute units (ten-fold for in visceral and four-fold in subcutaneous adipose tissue). Likewise, macrophages decreased in visceral but not in subcutaneous adipose tissue (only appreciable by relative units). Its proinflammatory phenotypic subset (identified by CD11c) also decreased in both tissue depots after weight loss. B-lymphocytes were residual (around 1%), whereas T-lymphocytes were one of the principal populations in all groups (from 25% to 30%). The abundance of T-lymphocytes did not change when using relative units. Moreover, the increase observed with absolute units was not sufficient to compensate the mass reduction of adipose tissue after weight loss. Concerning the two main phenotypic subsets of T-cells, T-helper (CD4+) and T-cytotoxic (CD8+), we observed no differences between patient cohorts.

### SVF composition between visceral and subcutaneous adipose tissue

The physiological differences between the two fat depots analysed in this study are reflected in quantity and phenotypic composition of their SVF. The most remarkable feature of visceral adipose tissue is the abundance of total SVF, which was several folds greater than subcutaneous tissue (the average increase was 4.25 ± 1.56). As a result, the density (cell/g) of almost all SVF populations was higher in visceral adipose tissue from both cohorts (Table 3). Within the phenotypic spectrum of macrophages, the CD11c+ proinflammatory subset in morbidly obese patients increased in the subcutaneous depot. Interestingly, within T-cells, the proportion of CD4+ was more elevated in subcutaneous adipose tissue, without regard of patient cohort (Table 3).

### Multivariate analysis

SVF populations were expressed in relative units because of the influence of adipocyte volume on mass tissue. However, the normalization of data in relation to a reference population increases the collinearity among variables. Therefore, our model analyses the relationship among AdPCs and the phenotype of immune cells (proportion of CD11c within macrophages and proportion of T-cytotoxic within T-lymphocytes) in the context of surgery-induced weight loss (Table 4). In visceral adipose tissue, AdPCs is the only independent variable associated with weight reduction. On the other hand, in subcutaneous adipose tissue, AdPCs increases in relation to weight loss with independence of sex and age, but depending on the phenotype of macrophages.

## Discussion

White adipocyte is a terminally differentiated postmitotic cell. Consequently, the only way to generate new adipocytes is through the activation of self-renewing AdPCs. Initial studies have suggested that adipocyte number is fixed at the beginning of adulthood^22^. According to this concept, in adult subjects, adipose tissue would expand preferably because of adipocyte hypertrophy, while AdPCs activation would be restricted to the reposition of death adipocytes^22^. Although the issue is not completely resolved^23^, further works reported that cell hyperplasia enlarges adipose tissue during adulthood^12,13^. Indeed, the reservoir of AdPCs contained in fat depots is exceptionally high^17^, thus suggesting that adipose tissue enlargement requires a strong responsiveness by adipocyte hyperplasia in order to properly react to a sustained stimulus of adipogenesis. However, it is believed that the contraction of body fat mass after weight loss is produced by reduction of fat cell volume without significant decrease of adipocyte number^24^. In this regard, specific mechanisms for adipocyte elimination after weight loss have not yet been described.

SVF isolated from adipose tissue has showed *in vitro* potentiality to differentiate into mesoderm lineage cells and, according to certain reports, into some ectoderm and endoderm cell types^25^. Therefore, several terms such as “adipose tissue-derived stem cells” (ASCs) have been coined. However, in contrast to bone marrow-derived mesenquimal stem cells, in which there is an international consensus about the pattern of surface cell markers^26^, agreement for a definitive ASC marker profile remains to be established. Adipogenesis is considered the most relevant potential *in vivo* within ASCs^25,27^. The cell marker profile that identifies the adipogenic potential has been characterized in mouse SVF^19^ but persists to be defined in humans. Taking into account this problem, we have considered the CD45−/CD31−/CD34+ cytometric fraction (most commonly used in literature) as representative of the ensemble of progenitor cell populations which contains the adipogenic potential in humans SVF^28^. It must be noted that a small proportion of non-adipogenic cells should be assumed within this marker profile. At this point, let us remark some terminological imprecision in the scientific literature. The term “preadipocyte”, depending on the author, defines the set of progenitor cells highly committed with the adipocyte lineage, or the whole of the progenitor populations with some adipogenic potential^20,25^. Moreover, ASCs, preadipocytes or even the crude SVF have sometimes been used as synonyms^29,30^.

It is well documented that obesity-related pathologies are associated with increased adipocyte hypertrophy, regardless of BMI^21,31,32^. According to the adipose tissue expandability hypothesis, in a context of hypercaloric diet, the difficulty of continued expanding adipose tissue promotes ectopic accumulation of lipids and the development of obesity-related pathologies^9^. In accordance with a previous published study^21^, we have observed that AdPCs quantity decreases in morbidly obese patients with type II diabetes (Table 2). These results suggest that adipocyte hyperplasia could be compromised below a certain threshold of progenitor cells. Furthermore, apart from the size of the AdPC reservoir, other factors can determine the hyperplastic potential of adipose tissue, including chronic disorders impairing control of adipocyte differentiation (such as chronic inflammation or endocrine resistances)^33,34^. It should be noted that multiple populations coexist within the concept of AdPCs, with notable differences in the level of commitment to the adipogenic cell fate^19,27,35,36^. Interestingly, the phenotypic diversity of AdPCs depends on physiological factors, body fat localization and sexual determinants^35–37^. This suggests that the hyperplastic potential is controlled by a complex dynamic population of tissue progenitor cells.

In this study, we report a notable increment of the AdPCs reservoir after a period of sustained weight loss (Table 3, Fig. 4). This increase supports the idea of an autonomous mechanism that rises the quantity of progenitor cells during the adipose tissue remodelling. Formally, we cannot conclude whether or not this increment tends to restore a normal hyperplastic potential in an adipose tissue that is recovering its homeostatic functions. This hypothesis should be explored in future studies.

We found a higher density of endothelial cells in visceral adipose tissue (Table 3), which is coherent because there is more vascularization at visceral fat depots^38^, but we only observed a moderate increase after losing weight in both types of adipose tissue (Table 3, Fig. 4). We have also found that the magnitude of endothelial cells (cell/g of tissue) is comparable to that estimated by Villaret *et al*.^38^. This study quantified the endothelial network of adipose tissue by confocal immunofluorescence analysis of CD34+/CD31+ cells. In fact, endothelial cells are usually detected by the simultaneous expression of CD34 and CD31^39^. However, some tissues can show different expression patterns^39^. Moreover, certain authors considered that, in adipose tissue, mature endothelial cells on capillaries are marked by CD34−/CD31+^40^. Notwithstanding the foregoing, in our study almost all CD45−/CD31+ events showed a low- or medium-expression level for CD34. Only in some samples we found a small population CD45−/CD34−/CD31+ (Fig. 2). In our opinion, this controversy is originated in the phenotypic diversity of endothelial cells as well as in methodological differences.

It is widely accepted that obesity is associated with a state of low-level chronic inflammation at adipose tissue, which increases its intensity as the patient progress in obesity-related pathologies^10^. In this context, a variable but elevated proportion of SVF is composed by tissue-resident immunological cells belonging to both, innate and adaptive immune system. The phenotypic diversity, displayed by these immune cell populations, changes depending on diverse factors such as body fat localization, inflammation or tissue remodelling processes^10,11,41^.

Adipose tissue macrophages, the best-known immune population, have been studied since 2003. The first article published described proinflammatory phenotypic switching in obese animals^42^. In parallel, obesity leads the increase of macrophages by two mechanisms, monocyte recruitment and local proliferation^42,43^. Our cytometric panel includes CD11c as a phenotypic marker. Although a single marker cannot determine the phenotypic spectrum of macrophages, CD11c is considered an appropriate representative of pro-inflammatory M1 phenotypes^44^. We showed a decrease in the proportion of CD11c+ macrophages after weight loss (Table 3) which, according to literature^44^, suggests a shift towards the predominance of non-proinflammatory phenotypes.

Neutrophils were the most important myeloid infiltration in morbidly obese patients. In agreement with previously published works^45^, our data suggest that neutrophil quantity reflects the existence of an inflammation source at adipose tissue, which decreased after losing weight (Table 3, Fig. 4). However, we must assume that a variable and unknown proportion of neutrophils may have artificially infiltrated adipose tissue owing to surgery-induced acute inflammation^46^. In addition, acute inflammation might be affected in different ways by the distinct surgical procedures carried out in our patients, which increases the difficulty to analyse adipose tissue neutrophils.

B and T-lymphocytes have been regarded as components of the chronic inflammatory environment (developed at fat depots during obesity^47^). Nevertheless, the paradigm is being redefined. Adipose tissue begins to be considered as an immunological organ, closely associated with the immune network. In 2010, Moro *et al*. described the existence of lymphoid structures into adipose tissue (Fat-Associated Lymphoid Clusters)^48^ with a high density of B- and T-lymphocytes. However, T-lymphocytes are also abundant outside FALC areas^49^. The distribution of FALCs is heterogeneous among different fat depots – very abundant in pericardial adipose tissue but scarce in omental and, particularly, in subcutaneous depots^49^. Interestingly, several recent works have showed that white adipose tissue is a major reservoir of memory T-lymphocytes, with the ability to arm autonomous immunological responses against infections^50^. In our study, B-lymphocytes appeared to be residual, probably as consequence of low density of FALC areas at omental and subcutaneous adipose tissues. More interesting is the dynamic between the two main T cell phenotypes, T-helper and T-cytotoxic. T-helper lymphocytes were more abundant in subcutaneous adipose tissue without regard to the patient cohort (Table 3). This suggests that adipose tissue T cells are exposed to different antigen collections depending on the localization of body fat depots. The current challenge lies in discerning the collection of antigens against which infiltrated lymphocytes are reactive and the footprint that obesity may leave on the TCR repertoire of adipose tissue memory T-lymphocytes.

We report a neat increase in the quantity of mast cells in adipose tissue after weight loss (Table 3, Fig. 4). The roll of mast cells in the pathophysiology of adipose tissue is unclear, and contradictory results can be found in the scientific literature^51–53^. More studies are necessary to elucidate the tissue function of mast cells, focusing on the phenotypic diversity and the production of cytokines.

Our methodological approach permits the simultaneous quantification of many different cell types and, as a result, we have been able to obtain an overall view of the main cell populations present at adipose tissue, in two different physiological situations. Nevertheless, we must assume certain methodological limitations at the SVF isolation level. A proportion of the lipid-filled macrophages (which clear death adipocytes in crown-like structures^54^) could not pellet and may remain in the supernatant with the adipocytes. Moreover, it is possible that, in the ex-morbidly obese cohort, some mature adipocytes could have considerably reduced the size of their lipid droplet. These adipocytes may increase its density coefficient and could pellet with the SVF. This eventuality is not relevant inasmuch as our criteria to identify SVF cells during the cytometry analysis (CD31+, CD34+ or CD45+) excludes contaminant mature adipocytes. However, several subsets of pericytes (negatives for CD34 and CD31) which present some adipogenic potential^40^, are not identified by our methodological design, since we cannot discern between CD31−/CD34−/CD45− cells and tissue debris.

To the best of our knowledge, we report for the first time that, in obese patients, the quantity of AdPCs and mast cells considerably increase after a sustained period of losing weight. In parallel, we observed a further decrease in the quantity of neutrophils and a phenotypic switching of macrophages. These changes were produced in the two different fat depots studied. However, no variations were observed for lymphocytes, neither in quantity nor in T-helper proportion. We believe our work is useful for future studies of the mechanisms underlying tissue remodelling triggered by weight loss, its repercussion on the reversion of obesity-related pathologies and the possible existence of a point of no return.

## Data Availability

The datasets generated during and/or analysed during the current study are available from the corresponding authors on reasonable request.
